# Supplementary material for: CSB promoter downregulation via histone H3 hypoacetylation is an early determinant of replicative senescence
Source: Nat Commun. 2019 Dec 6;10:5576. doi: 10.1038/s41467-019-13314-y (PMC6898346; doi:10.1038/s41467-019-13314-y)
Supplement: Supplementary file 1 — Supplementary Information [file 41467_2019_13314_MOESM1_ESM.pdf]

Supplementary Information

CSB promoter downregulation via histone H3 hypoacetylation is an early determinant of replicative senescence

Crochemore et al.

Supplementary Figure 1

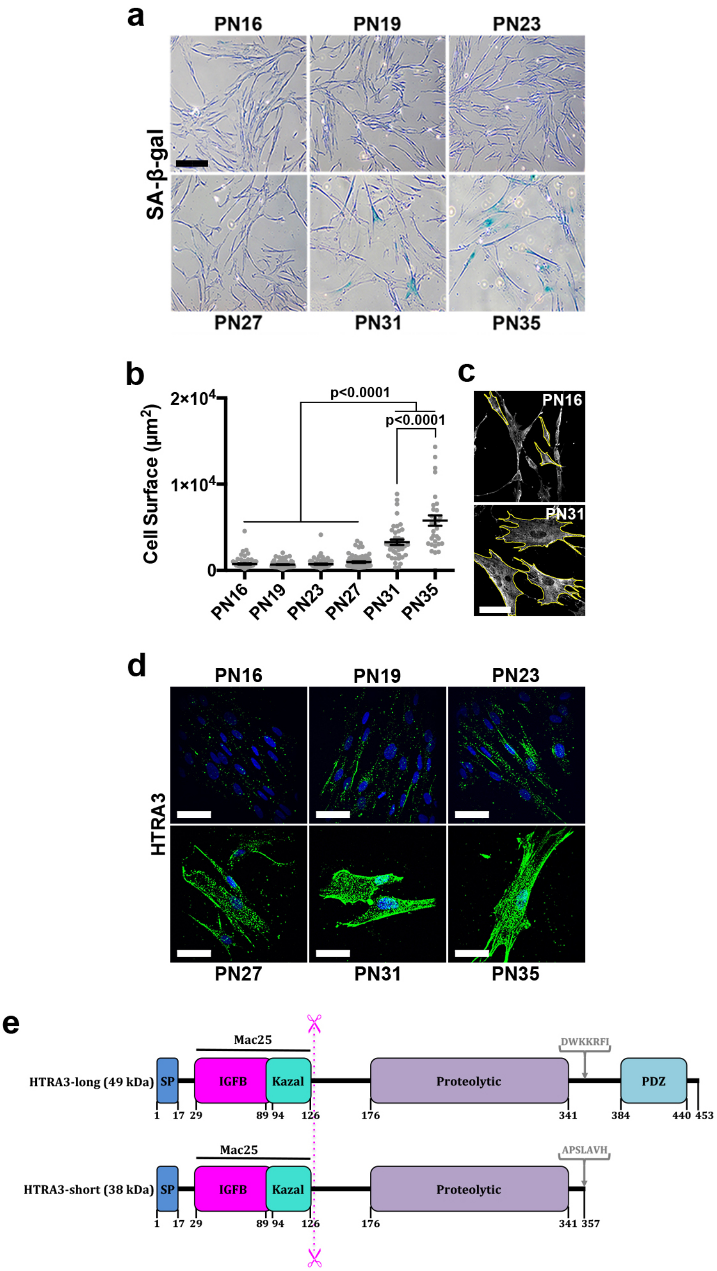

Assessment of senescence parameters and HTRA3 in IMR-90 fibroblasts

(a) Representative images of SA-β-gal$^+$ cells at various PNs, quantified in Fig 1b; Scale bar = 200µM. **(b)** Quantification of cell surface and **(c)** representative images of cell contours (contrast of immunomarker in panel_d). n=30-80 cells from 3 independent experiments, mean ± SEM; one-way ANOVA (F=113.2, DFn=5, DFd=383, p<0.0001) with post-hoc Tukey's test. **(d)** Representative confocal acquisitions of cells immunostained for HTRA3 (green) and counterstained with Hoechst (blue, nuclei) after maximum intensity projection with Imaris software. Scale bar= 50µM. **(e)** Schematic subdomains description of the HTRA3 long (49 kDa) and short (38 kDa) isoforms, generated by alternative splicing[1]. From the N-terminus both isoforms are composed of the signal secretory peptide (SP), an insulin-like growth factor binding domain (IGFB), and a Kazal type inhibitor motif (Kazal) that shares homology with the Mac25 protein (IGFBP7), a secreted protein that binds insulin-like growth factors, and a proteolytic trypsin-like domain (Proteolytic). The long isoform is additionally composed of a PDZ domain (PDZ) that mediates protein-protein interaction. The short isoform ends with an aminoacid sequence (APSLAVH) that differs form the corresponding aminoacids of the long isoform (DWKKRFI). Numbers represents first and last aminoacid of each subdomain. The magenta dotted line represents an identified auto-cleavage site, although additional autocleavage sites may exist [2]. Source data are provided as Source Data files.

Supplementary Figure 2

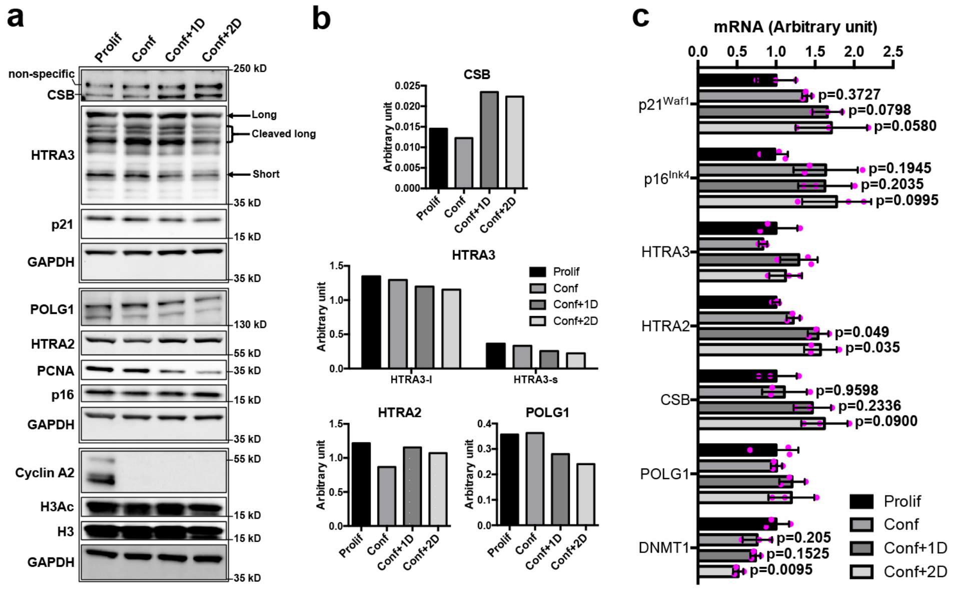

Proliferation-independent deregulation of HTRA3 and CSB during senescence

(a) Immunoblot of PCNA, Cyclin A2, p21, p16, HTRA3, HTRA2, CSB, POLG1, acetylated histone H3 and total histone H3, from whole-cell extracts of proliferative and slowly dividing/non-dividing IMR-90. F-C staining for HTRA2 and PCNA. Samples on the same blot are framed; each frame displays the respective GAPDH (F-C staining) used as a loading control. Early passage (PN18) IMR-90 were harvested at a proliferative stage of 70-80% (sub)confluency (Prolif) or at 100% confluency (Conf). For proliferation arrest, confluent cells were further incubated for either 1 day (Conf+1D) or 2 days (Conf+2D) before harvesting. The upper band in the CSB blot is considered non-specific since it is present in CSB-KO cells, despite its levels decrease after CSB deletion[3]. **(b)** Corresponding quantification of HTRA3, HTRA2, CSB, and POLG1 immunoblot band intensity normalized to the respective GAPDH. **(c)** Quantitative RT-qPCR showing levels of $p21^{Waf1}$, $p16^{Ink4}$, *HTRA3, HTRA2*, *CSB*, *POLG1,* and *DNMT1* transcripts of proliferative or slowly dividing/non-dividing IMR-90. n=3 independent experiments, mean ± SD; one-way ANOVA ($p21^{Waf1}$: F=4.010, DFn=3, DFd=8, p=0.0516; $p16^{Ink4}$: F=2.946, DFn=3, DFd=8, p=0.0985; *HTRA3*: F=2.532, DFn=3, DFd=8, p=0.1305; *HTRA2*: F=12.48, DFn=3, DFd=8, p=0.0022; *CSB*: F=3.455, DFn=3, DFd=8, p=0.0714; *POLG1*: F=0.7844, DFn=3, DFd=8, p=0.5354; *DNMT1*: F=6.556, DFn=3, DFd=8, p=0.0151) with post-hoc Tukey's test *vs.* Prolif. The confluent condition (massive cell detachment during the procedure) did not allow assessing the number of SA-β-gal-positive or SA-β-gal-negative cells. Source data are provided as Source Data files.

Supplementary Figure 3

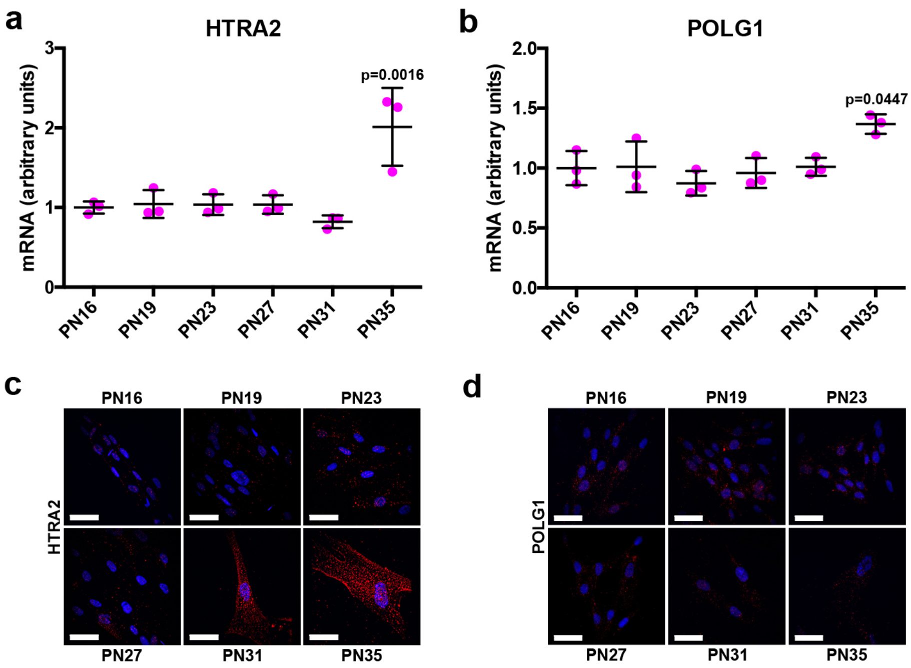

Assessment of HTRA2 and POLG1 during senescence in IMR-90 fibroblasts

RT-qPCR of **(a)** *HTRA2* and **(b)** *POLG1* in IMR-90. n=3 independent experiments, mean ± SD; one-way ANOVA (*HTRA2*: F=10.48, DFn=5, DFd=12, p=0.0005; *POLG1*: F=5.036, DFn=5, DFd=12, p=0.0102) with post-hoc Tukey's test *vs.* PN16. Representative confocal acquisitions of cells immunostained for **(c)** HTRA2 (red) and **(d)** POLG1 (red), counterstained with Hoechst (blue) after maximum intensity projection with Imaris; scale bar= 50μM. Quantification is shown in Fig. 1g (HTRA2) and Fig. 1h (POLG1). Source data are provided as Source Data files.

Supplementary Figure 4

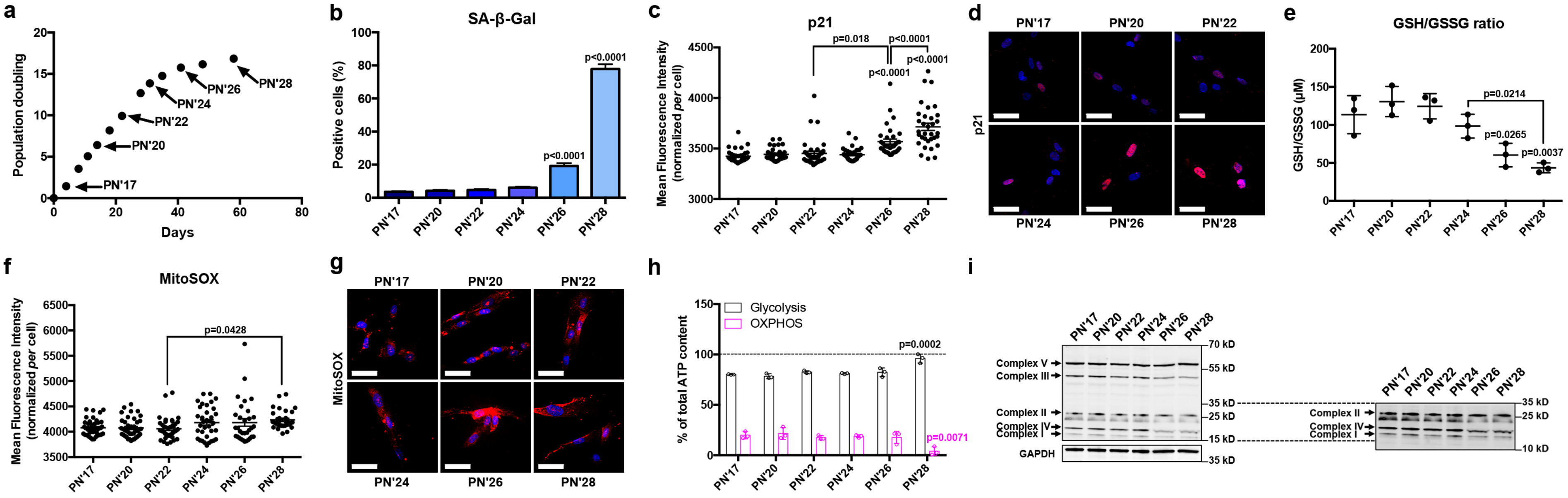

Reduced mitochondrial complexes and glycolytic shift during replicative senescence

(a) Growth curve of serially passaged IMR-90, expressed as cumulative population doubling. Cells in this figure started from IMR-90 fibroblasts thawed at PN15, which underwent one more thawing episode than experiments performed elsewhere in this study, *i.e.* cells thawed at PN14 (Figs 1-5, 6a, 7a, and, Supplementary Figs. 1-3, 5, 9[except c-f]-10). Senescence corresponds to the plateau (proliferative arrest). Cells have been analysed at various passages (black arrows), and identified by the respective PN' (to distinguish from experiments in Figs 1-5, 6a, 7a, and, Supplementary Figs. 1-3, 5, 9 [except c-f]-10). In this experiment the end of the exponential phase was reached at PN'24, pre-senescence at PN'26, and senescence at PN'28. n=3 independent cultures; mean ± SD, values are reported in Source Data files **(b)** Percent of cells positively stained for SA-β-gal; n=1010-1210 cells (PN'17-PN'24) and n=200-480 (PN'26-PN'28) from 3 independent experiments, mean ± SEM; one-way ANOVA (F=374.1, DFn=5, DFd=5091, p<0.0001) with post-hoc Tukey's test *vs.* PN16. **(c)** Quantification of p21 (senescence marker); n=30-50 cells from 3 independent experiments, mean ± SEM, and **(d)** representative confocal acquisitions of cells immunostained for p21 (green) and counterstained with Hoechst (blue, nuclei) after maximum intensity projection with Imaris. Scale bar= 50μM. **(e)** GSH/GSSG ratio to measure oxidative stress; n=3 independent experiments, mean ± SD. **(f)** Quantification of MitoSOX (a marker of mitochondrial ROS) mean fluorescence intensity per cell, at the indicated PN', n=30-50 cells from 3 independent experiments, and **(g)** representative confocal acquisitions of cells treated with MitoSOX (green) and counterstained with Hoechst (blue, nuclei) after maximum intensity projection with Imaris. Scale bar= 50μM. mean ± SEM. In c-f; one-way ANOVA (panel_c): F=29.02, DFn=5, DFd=205, p<0.0001; (panel_e): F=12.58, DFn=5, DFd=12, p=0.0002; (panel_f): F=3.076, DFn=5, DFd=218, p=0.0105) with post-hoc Tukey's test *vs.* PN'17 (p-values on the top of columns) when not specified. **(h)** Fractions of glycolytic (oligomycin-insensitive, black-framed) and OXPHOS (oligomycin-sensitive, magenta-framed)-dependent ATP content in IMR-90 from PN'17 to PN'28. Mean ± SD from 3 independent experiments; SD of glycolytic and OXPHOS-dependent ATP are indicated with the same colour code as the respective columns; one-way ANOVA (Glycolysis, in black: F=15.22, DFn=5, DFd=12, p<0.0001; OXPHOS, in magenta: F=6.546, DFn=5, DFd=12, p=0.0037) with post-hoc Tukey's test *vs.* the respective PN'17. **(i)** Immunoblot of the five mitochondrial respiratory complexes and GAPDH (F-C staining) loading control from whole-cell extracts of IMR-90 at the indicated PNs. On the right, increased contrast for the portion of the blot displaying factors of complexes I, II, and IV. Source data are provided as Source Data files.

Supplementary Figure 5

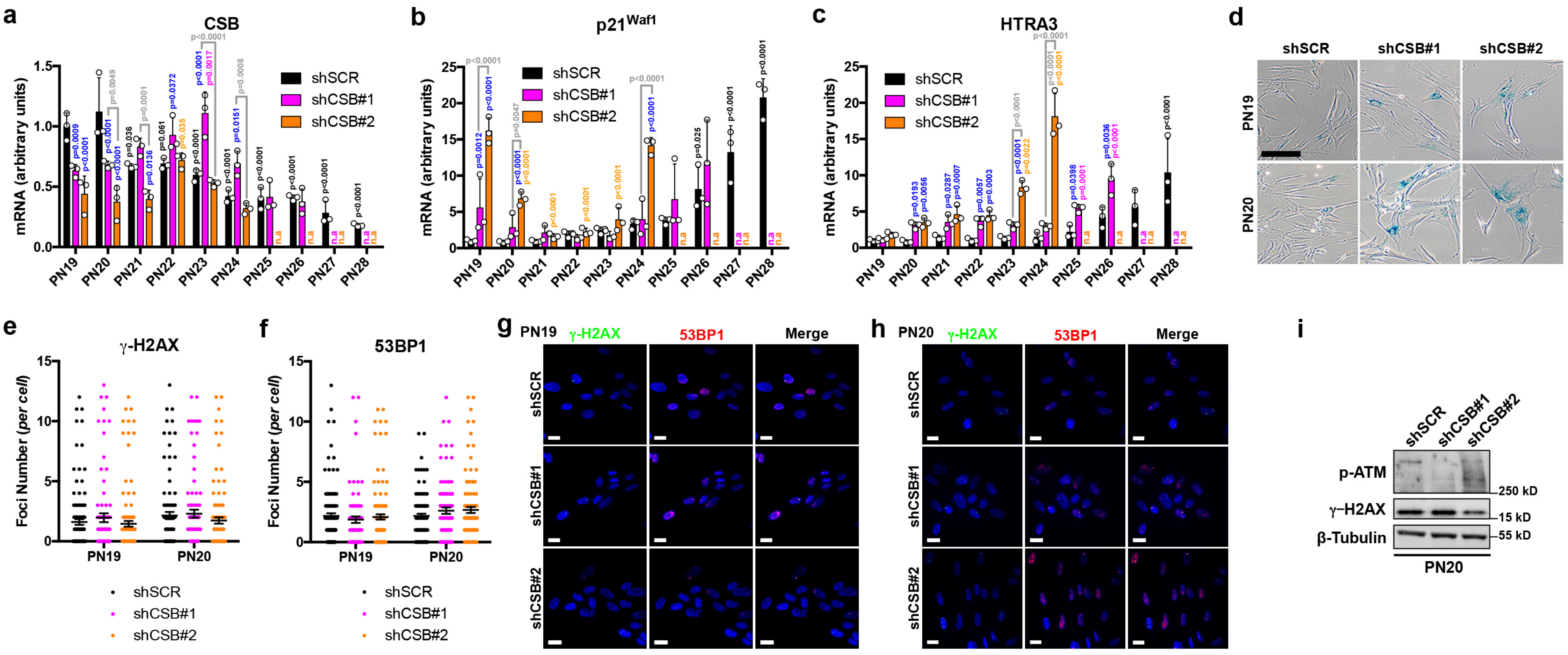

Transient knockdown of CSB anticipates induction of senescent markers $p21^{Waf1}$ and HTRA3

mRNA expression of **(a)** *CSB*, **(b)** *$p21^{Waf1}$*, and **(c)** *HTRA3* assessed by RT-qPCR in IMR-90 knocked down for *CSB* (shCSB#1 and shCSB#2) or scramble control (shSCR) at the indicated PN. n=3 independent experiments, mean ± SD; two-way ANOVA (*CSB*: F=49.38, DFn=2, DFd=36, p<0.0001 (PN19-24); F=0.06430, DFn=1, DFd=8, p=0.8062 (PN25-26). *$p21^{Waf1}$*: F=79.85, DFn=2, DFd=36, p<0.0001 (PN19-24); F=1.970, DFn=1, DFd=8, p=0.1980 (PN25-26). *HTRA3*: F=162.8, DFn=2, DFd=36, p<0.0001 (PN19-24); F=27.99, DFn=1, DFd=8, p=0.0008 (PN25-26)) with post-hoc Tukey's (PN19-24) or Sidak's (PN25-26) tests, *vs.* shSCR at each PN (blue p-values), or shCSB#1 *vs.* shCSB#2 (grey p-values); one-way ANOVA (*CSB*: F=22/96, DFn=9, DFd=20, p<0.0001 (shSCR); F=14.76, DFn=7, DFd=16, p<0.0001 (shCSB#1); F=6.752, DFn=5, DFd=12, p=0.0033 (shCSB#2). *$p21^{Waf1}$*: F=41.92, DFn=9, DFd=12, p<0.0001 (shSCR); F=3.428, DFn=7, DFd=16, p=0.0196 (shCSB#2); F=79.76, DFn=5, DFd=12, p<0.0001 (shCSB#2). *HTRA3*: F=8.817, DFn=9, DFd=20, p<0.0001 (shSCR); F=20.58, DFn=7, DFd=16, p<0.0001 (shCSB#1); F=46.88, DFn=5, DFd=12, p<0.0001 (shCSB#2)) with post-hoc Tukey's test for each group, *vs.* the corresponding PN19 (black p-values for shSCR, red p-values for shCSB#1, and orange p-values for shCSB#2). n.a; not applicable (no surviving cells at the corresponding passages). PN19 and PN20 from these experiments are individually represented in Figs. 3c, g, h. *HTRA3* and *$p21^{Waf1}$* levels increased biphasically, a first time following CSB downregulation (at PN20 and PN19, respectively) and a second time when CSB decreased "physiologically" during cellular senescence (at PN24 or later). **(d)** Representative images of the SA-β-gal staining quantified in Fig 3f; Scale bar= 200μM. Quantification of the foci number *per* cell of IMR-90 fibroblasts immunostained for **(e)** γ-H2AX (green) and **(f)** 53BP1 (red) and counterstained with Hoechst after maximum intensity projection with the Imaris software, and representative confocal acquisitions at **(g)** PN19 and **(h)** PN20 upon CSB knock down (shCSB#1 and shCSB#2) or shSCR; Scale bar= 20μM. Quantification from 3 independent experiments, n=90-127 cells/condition. All data set comparisons are not significant (two-way ANOVA (γ-H2AX: F=1.742, DFn=2, DFd=617, p=0.1761; 53BP1: F=0.4430, DFn=2, DFd=617, p=0.6423) with post-hoc Tukey's test). **(i)** Immunoblots of the DNA damage response proteins p-ATM and γ-H2AX and the housekeeping protein b-tubulin at PN20. Source data are provided as Source Data files.

Supplementary Figure 6

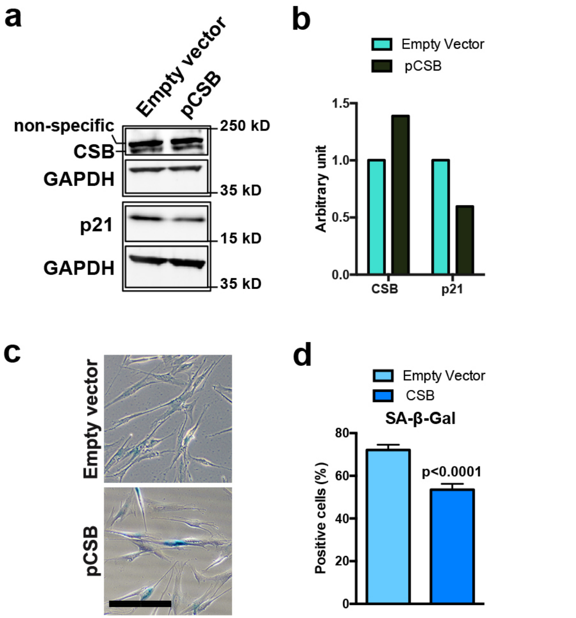

Ectopic overexpression of CSB delays induction of senescent markers SA-β-gal and p21^{Waf1}

(a) WB of CSB and p21, and the respective GAPDH as a control and **(b)** corresponding quantification normalized to GAPDH. **(c)** Representative images (Scale bar= 200μM) and **(d)** quantification of SA-β-gal^{+} cells. n=333 cells from 3 independent experiments, mean ± SEM; unpaired Student's t-test (two-tailed) (t=5.059, DF=664), p-value *vs*. Empty vector. Source data are provided as Source Data files.

Supplementary Figure 7

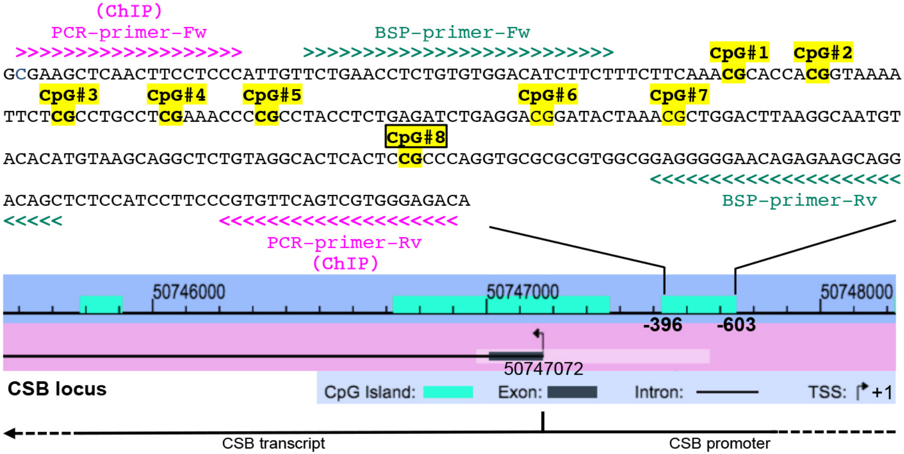

CSB promoter region of interest

Schematic representation of the CSB promoter region (Chr10:50746446-Chr10:50747847 (Ensembl GRCh37 coordinates))[4]. In the CSB promoter, the tested region (from -396 to -603 of the TSS (transcription start site) Chr10:50747072), is a CpG island (green) in which we analysed the level of methylation of eight CpG sites (yellow) using bisulfite sequencing PCR (BSP) primers (green arrows). These CpG sites are located at -564 (CpG#1), -557 (CpG#2), -545 (CpG#3), -536 (CpG#4), -528 (CpG#5), -505 (CpG#6), -494 (Cpg#7), -441 (Cpg#8) from the TSS. The CpG#8 (framed in black) is hypermethylated in a pathological condition (age-related nuclear cataract)[4]. The same promoter region was targeted to assess the level of histone H3 acetylation by ChIP using PCR primers (red arrows). Fw (forward) and Rv (reverse) primers of each pair are indicated. The graphical representation the CSB locus was adapted from urogene.org.

Supplementary Figure 8

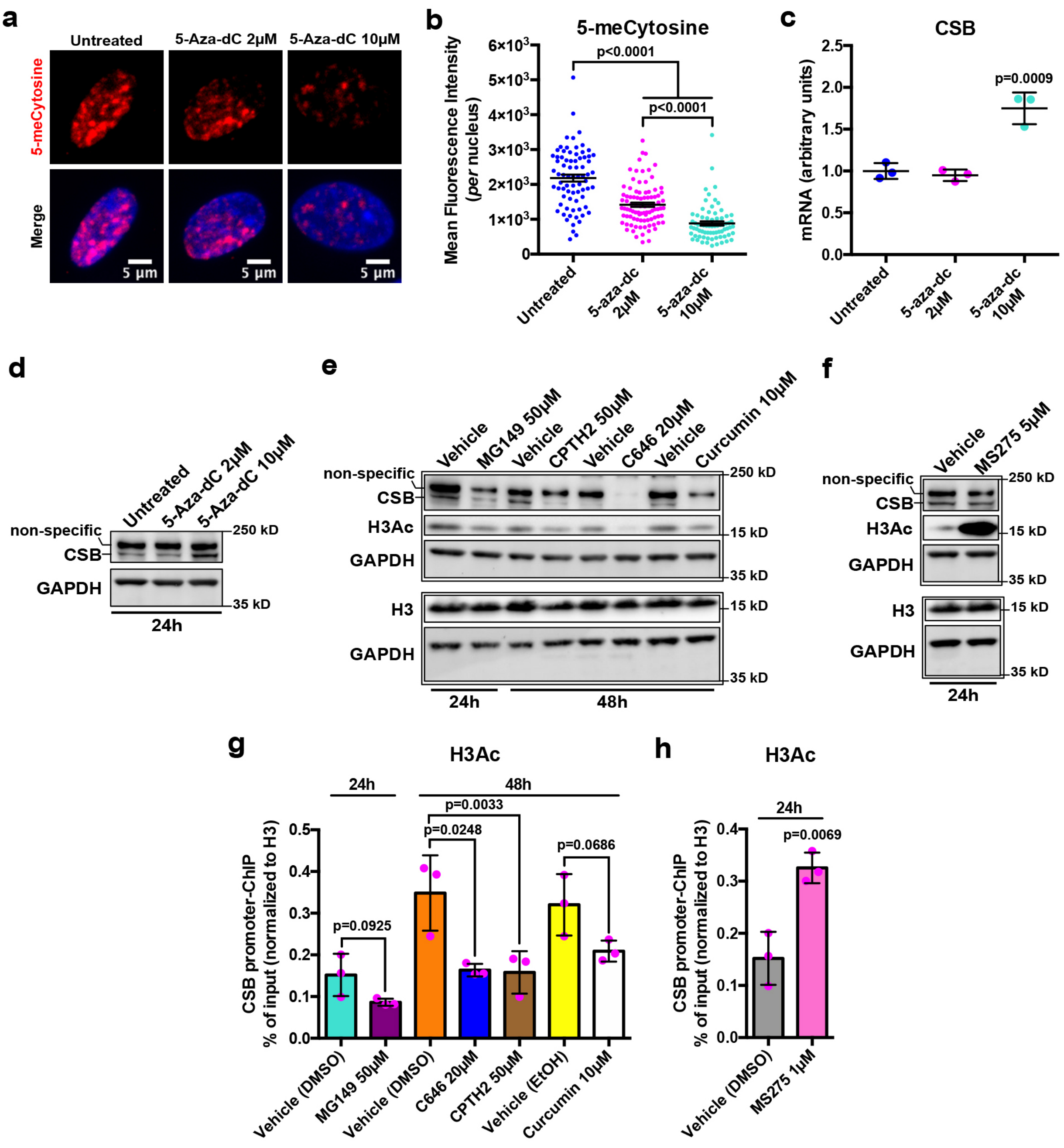

Epigenetic modifications at the global level and at the CSB promoter

(a) Representative confocal acquisitions of IMR-90 fibroblasts immunostained for 5-meCytosine (red) and counterstained with Hoechst after maximum intensity projection with the Imaris software in the presence and in the absence of the methyltransferase inhibitor 5-aza-dC (scale bar= 20μM), and **(b)** quantification from 3 independent experiments; n=73-91 cells/condition; one-way ANOVA (F=73.09, DFn=2, DFd=236, p<0.0001) with post-hoc Tukey's test. **(c)** RT-qPCR of *CSB* in IMR-90 in the presence (two concentrations) and in the absence of 5-aza-dC. n=3 independent experiments, mean ± SD; one-way ANOVA (F=36.23, DFn=2, DFd=6, p=0.0004) with post-hoc Tukey's test *vs.* untreated; p-value on top of the scatter plot. **(d)** WB of CSB with GAPDH used as a loading control. In b-d, 2μM and 10 μM 5-aza-dC were used. WB of CSB, acetylated histone H3, histone H3 **(e)** after treatment with different HAT inhibitors, namely MG149 (a anacardic acid derivative with sensitivity toward Tip60 and MOF HATs, which are also involved in DNA repair), CPTH2 (specific of Gcn5p HAT), C646 (a potent selective inhibitor of p300 HAT), or curcumin (specific of p300/CBP), and **(f)** with the HDAC inhibitor MS275, specific of class I HDAC; samples on the same blot are framed; each frame displays the respective GAPDH used as a loading control. **(g)** Quantitative PCR analysis of a DNA fragment in the CSB promoter from ChIP assay with α-H3 acetylated in the presence and in the absence of HAT inhibitors, and **(h)** of the HDAC inhibitor MS275; n=3 independent experiments, mean ± SD; unpaired Student's t-test (two-tailed) (MG149: t=2.201, DF=4; C646: t=3.506, DF=4; CPTH2: t=3.188, DF=4; Curcumin: t=2.475, DF=4; MS275: t=5.115, DF=4), p-values *vs.* the corresponding Vehicle**.** Source data are provided as Source Data files.

Supplementary Figure 9

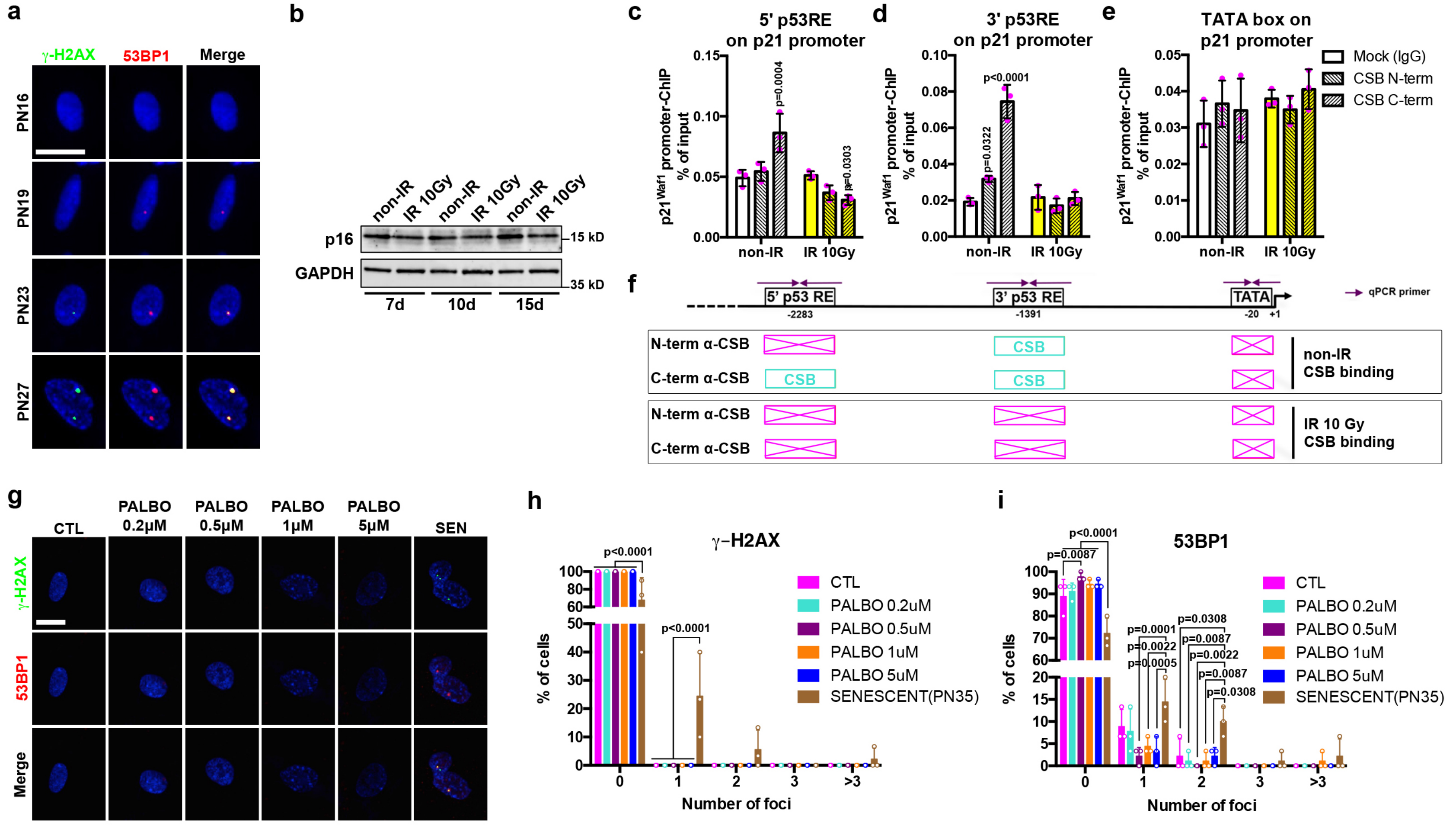

Presence of DNA damage in replicative senescence and absence of DNA damage in palbociclib-induced senescence

(a) Representative confocal acquisitions of IMR-90 fibroblasts during exponential growth (PN16-PN27) immunostained for γ–H2AX (green) and 53BP1 (red) and counterstained with Hoechst after maximum intensity projection with the Imaris software, quantified in Fig. 7a; scale bar= 20μM. **(b)** WB of p16 at different time points post-IR; GAPDH used as a loading control. **(c, d, e)** Quantitative PCR of three distinct DNA fragments in the *p21*^{Waf1} promoter from ChIP assays with either N-term α-CSB [Bethyl] or C-term α-CSB [Abcam] antibodies in non-irradiated and irradiated (10 Gy) cells; n=3 independent experiments; for each tested region two-way ANOVA (5'p53RE: F=3.628, DFn=2, DFd=12, p=0.0586. 3'p53RE: F=46.57, DFn=2, DFd=12, p<0.0001. TATA: F=0.4303, DFn=2, DFd=12, p=0.6599.) with post-hoc Tukey's test *vs.* the corresponding Mock. **(f)** Scheme not at scale of primer positions on the p21 promoter and summary of positive amplifications in turquoise (boxes in magenta indicate no amplification). **(g)** Representative confocal acquisitions of palbociclib-treated (0.2, 0.5, 1 or 5μM) and untreated IMR-90 fibroblasts immunostained for γ-H2AX (green) and 53BP1 (red) and counterstained with Hoechst (blue, to identify the nuclei) after maximum intensity projection with the Imaris software; scale bar= 20μM. Quantification of the percentage of cells containing 0, 1, 2, 3, or >3 **(h)** γ-H2AX and **(i)** 53BP1 *foci* per nucleus; these foci indicate DNA damage, essentially DSBs (double-strand breaks). Replicative senescent IMR-90 fibroblasts at PN35 were used as a positive control for γ-H2AX and 53BP1 foci. n=90 cells from 3 independent experiments. Mean ± SEM; two-way ANOVA (γ-H2AX: F=6.690, DFn=20, DFd=60, p<0.0001; 53BP1: F=9.587, DFn=20, DFd=60, p<0.0001) with post-hoc Tukey's test. Source data are provided as Source Data files.

Supplementary Figure 10

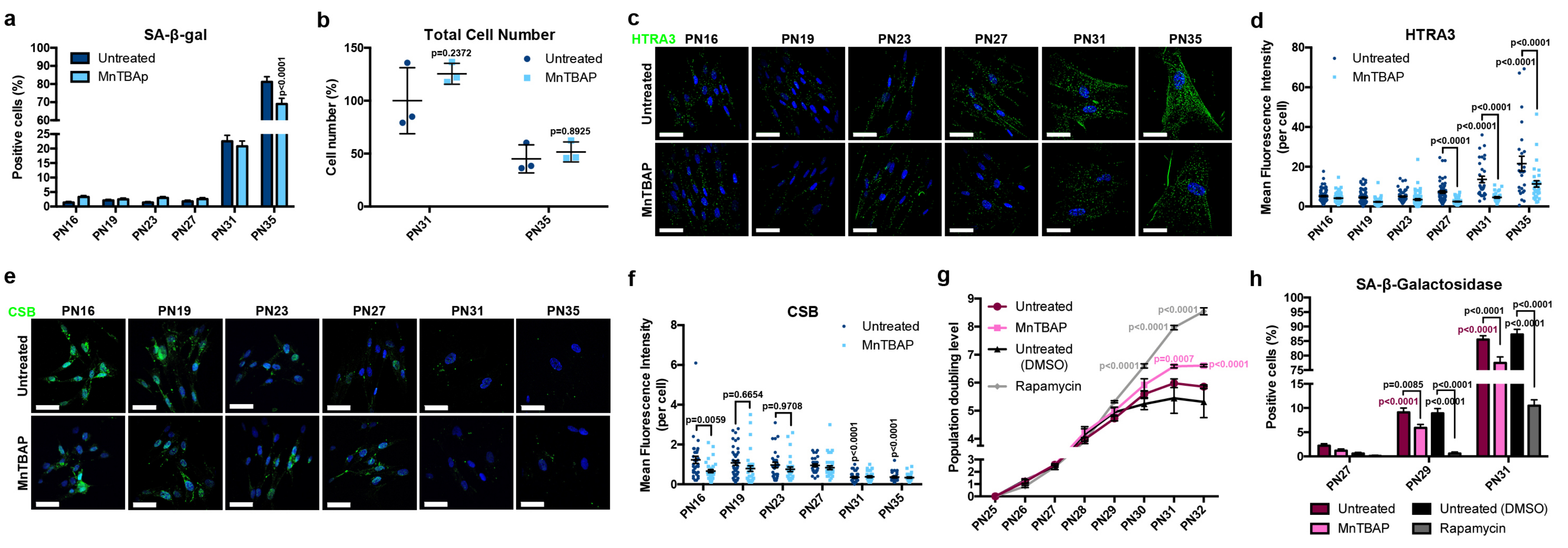

MnTBAP delays senescence

(a) Quantification of SA-β-gal^{+} cells. To perform the treatment in parallel for all PNs, fibroblasts analysed at each PN were obtained from cells thawed at the earlier PN. Fibroblasts at six PNs (from PN16 to PN35) were each individually treated with 100μM of MnTBAP or not (Untreated) for 24h. n=1050-1470 cells (PN16-PN27) and n=180-510 cells (PN31-PN35) from 3 independent experiments, mean ± SEM; two-way ANOVA (F=10.11, DFn=1, DFd=11286, p=0,0015) with post-hoc Tukey's test *vs.* the corresponding Untreated. **(b)** Total number of cell (SA-β-gal^{+} and SA-β-gal^{-}) in the presence and in the absence of MnTBAP, expressed as percentage of Untreated (100%); n=3 independent experiments; two-way ANOVA (F=2.297, DFn=1, DFd=8, p=0.1681) with post-hoc Sidak's test. Representative confocal acquisitions and quantification of HTRA3 **(c, d)** and CSB **(e, f)** IF in fibroblasts treated for 24h with MnTBAP or untreated, at the indicated PN. Cells were counterstained with Hoechst (blue, nuclei) and maximum intensity projection was applied using Imaris software. Scale bars = 50μM. n=30 cells from 3 independent experiments. Mean ± SEM; two-way ANOVA (HTRA3: F=13.15, DFn=5, DFd=733, p<0.0001; CSB: F=14.70, DFn=5, DFd=379, p<0.0001) with post-hoc Tukey's test *vs.* the corresponding PN16, when not specified. **(g)** Cumulative population doubling of serially passaged IMR-90 fibroblasts (starting at PN25, n=3 independent cultures). Cells were grown in the absence (Untreated) or in the presence of MnTBAP (10μM) or rapamycin (10nM, diluted in DMSO) in the culture medium, and kept in culture until they reach senescence (plateau). Mean ± SD; two-way ANOVA (F=100.5, DFn=3, DFd=64, p<0.0001) with post-hoc Tukey's test *vs.* Untreated (Pink) or *vs.* DMSO (Grey), p-values indicated with the same colour code as the respective curve. Quantification of **(h)** positively stained IMR-90 fibroblasts for SA-β-gal^{+}; n=370-1180 cells/condition from 3 independent experiments. Mean ± SEM; two-way ANOVA (F=527.3, DFn=3, DFd=9658, p<0.0001) with post-hoc Tukey's test *vs.* PN27 Untreated (Purple) or *vs.* PN27 DMSO (Black), when not specified. Source data are provided as Source Data files.

Supplementary Table 1

Gene/Region	Primer	Sequence (5′-3′)	Reference
Primers use for RTqPCR			
Human CSA	Forward	CGCCTTCGGAGAGCAGAGT	5
	Reverse	TGGATTCTTTCAACATCTCTG	5
Human CSB	Forward	CTGGAACAGGGAGTGCTTCA	6
	Reverse	ACTCCTTCTCCACGTCAACG	6
Human DNMT1	Forward	GCACAAACTGACCTGCTTCA	7
	Reverse	GCCTTTTCACCTCCATCAAA	7
Human DNMT3A	Forward	GACAAGAATGCCACCAAAGC	7
	Reverse	CGTCTCCGAACCACATGAC	7
Human DNMT3B	Forward	CCAGCTGAAGCCCATGTT	7
	Reverse	ATTTGTCTTGAGGCGCTTG	7
Human HDAC1	Forward	CTACTACGACGGGGATGTTGG	8
	Reverse	GAGTCATGCGGATTCGGTGAG	8
Human HDAC2	Forward	ATGGCGTACAGTCAAGGAGG	8
	Reverse	TGCGGATTCTATGAGGCTTCA	8
Human HTRA2	Forward	TTTGCCATCCCTTCTGATCG	9
	Reverse	ACACCATGCTGAACATCGGG	9
Human HTRA3-L	Forward	ATGCGGACGATCACACCAAG	9
	Reverse	CGCTGCCCTCCGTTGTCTG	9
Human HTRA3-S	Forward	GAGGGCTGGTCACATGAAGA	9
	Reverse	GCTCCGCTAATTTCCAGT	9
Human IL-6	Forward	AATTCGGTACATCCTCGACGG	10
	Reverse	GGTTGTTTTCTGCCAGTGCC	10
Human p16Ink4	Forward	GAAGGTCCCTCAGACATCCCC	11
	Reverse	CCCTGTAGGACCTTCGGTGAC	11
Human p21Waf1	Forward	GAGGCCGGGATGAGTTGGGAGGAG	11
	Reverse	CAGCCGGCGTTTGGAGTGGTAGAA	11
Human p53	Forward	GCGCACAGAGGAAGAGAATC	12
	Reverse	CTCTCGGAACATCTCGAAGC	12
Human POLG1	Forward	GAGAAGGCCCAGCAGATGTA	9
	Reverse	ATCCGACAGCCGATACCA	9
Human TBP	Forward	CTCACAGGTCAAAGGTTTAC	9
	Reverse	GCTGAGGTTGCAGGAATTGA	9
Primers used for qChIP			
CSB promoter	Forward	CGAAGCTCAACTTCCTCCC	This paper
	Reverse	GTCTCCCACGACTGAACACG	This paper
$p21^{Waf1}$ promoter;<br>3’ p53 response element	Forward	CTGTCCTCCCCGAGGTCA	13
	Reverse	ACATCTCAGGCTGCTCAGAGTCT	13
$p21^{Waf1}$ promoter;<br>5’ p53 response element	Forward	AGCAGGCTGTGGCTCTGATT	13
	Reverse	CAAAATAGCCACCAGCCTCTTCT	13
$p21^{Waf1}$ promoter; TATA	Forward	TATATCAGGGCCGCGCTG	13
	Reverse	GGCTCCACAAGGAACTGACTTC	13
Primers used for PCR-ChIP			
$p21^{Waf1}$ promoter;<br>3’ p53 response element	Forward	GAAATGCCTGAAAGCAGAGG	14
	Reverse	GCTCAGAGTCTGGAAATCTC	14
$p21^{Waf1}$ promoter;<br>distal p53 response element	Forward	GATGCCAACCAGATTTGCCG	14
	Reverse	CCTGGCTCTAACAACATCCC	14
Primers used for BSP			
CSB promoter	Forward	TGTTTTGAATTTTTGTGTGGATATTT	4
	Reverse	ACTATCCTACTTCTCTATTCCCCCTC	4

List of primers

References cited in the Table are indicated below.

Supplementary References

1. Beleford D, Rattan R, Chien J, Shridhar V. High temperature requirement A3 (HtrA3) promotes etoposide- and cisplatin-induced cytotoxicity in lung cancer cell lines. *J Biol Chem* **285**, 12011-12027 (2010).

2. Singh H, Makino S, Endo Y, Li Y, Stephens AN, Nie G. Application of the wheat-germ cell-free translation system to produce high temperature requirement A3 (HtrA3) proteases. *Biotechniques* **52**, 23-28 (2012).

3. Batenburg NL, Thompson EL, Hendrickson EA, Zhu XD. Cockayne syndrome group B protein regulates DNA double-strand break repair and checkpoint activation. *The EMBO journal* **34**, 1399-1416 (2015).

4. Wang Y, Li F, Zhang G, Kang L, Guan H. Ultraviolet-B induces ERCC6 repression in lens epithelium cells of age-related nuclear cataract through coordinated DNA hypermethylation and histone deacetylation. *Clin Epigenetics* **8**, 62 (2016).

5. Wang XF, Cui JZ, Nie W, Prasad SS, Matsubara JA. Differential gene expression of early and late passage retinal pigment epithelial cells. *Exp Eye Res* **79**, 209-221 (2004).

6. Lin Z*, et al.* A variant of the Cockayne syndrome B gene ERCC6 confers risk of lung cancer. *Hum Mutat* **29**, 113-122 (2008).

7. Bott AJ*, et al.* Oncogenic Myc Induces Expression of Glutamine Synthetase through Promoter Demethylation. *Cell Metab* **22**, 1068-1077 (2015).

8. Liu J*, et al.* Both HDAC5 and HDAC6 are required for the proliferation and metastasis of melanoma cells. *J Transl Med* **14**, 7 (2016).

9. Chatre L, Biard DS, Sarasin A, Ricchetti M. Reversal of mitochondrial defects with CSB-dependent serine protease inhibitors in patient cells of the progeroid Cockayne syndrome. *Proceedings of the National Academy of Sciences of the United States of America* **112**, E2910-2919 (2015).

10. Pfaffl MW. A new mathematical model for relative quantification in real-time RT-PCR. *Nucleic acids research* **29**, e45 (2001).

11. Yu Y*, et al.* Loss-of-function screening to identify miRNAs involved in senescence: tumor suppressor activity of miRNA-335 and its new target CARF. *Scientific reports* **6**, 30185 (2016).

12. Kalamegam G*, et al.* Pelleted Bone Marrow Derived Mesenchymal Stem Cells Are Better Protected from the Deleterious Effects of Arthroscopic Heat Shock. *Front Physiol* **7**, 180 (2016).

13. Laptenko O, Beckerman R, Freulich E, Prives C. p53 binding to nucleosomes within the p21 promoter in vivo leads to nucleosome loss and transcriptional activation. *Proceedings of the National Academy of Sciences of the United States of America* **108**, 10385-10390 (2011).

14. Saramaki A, Banwell CM, Campbell MJ, Carlberg C. Regulation of the human p21(waf1/cip1) gene promoter via multiple binding sites for p53 and the vitamin D3 receptor. *Nucleic acids research* **34**, 543-554 (2006).
