# Supplementary material for: CSB promoter downregulation via histone H3 hypoacetylation is an early determinant of replicative senescence
Source: Nat Commun. 2019 Dec 6;10:5576. doi: 10.1038/s41467-019-13314-y (PMC6898346; doi:10.1038/s41467-019-13314-y)
Supplement: Supplementary file 3 — Reporting Summary [file 41467_2019_13314_MOESM3_ESM.pdf]

nature research

Corresponding author(s): Miria Ricchetti 19-02448

Last updated by author(s): Oct 3, 2019

Reporting Summary

Nature Research wishes to improve the reproducibility of the work that we publish. This form provides structure for consistency and transparency in reporting. For further information on Nature Research policies, see Authors & Referees and the Editorial Policy Checklist.

Statistics

For all statistical analyses, confirm that the following items are present in the figure legend, table legend, main text, or Methods section.

n/a	Confirmed	
☐	☒	The exact sample size (*n*) for each experimental group/condition, given as a discrete number and unit of measurement
☐	☒	A statement on whether measurements were taken from distinct samples or whether the same sample was measured repeatedly
☐	☒	The statistical test(s) used AND whether they are one- or two-sided<br>*Only common tests should be described solely by name; describe more complex techniques in the Methods section.*
☒	☐	A description of all covariates tested
☐	☒	A description of any assumptions or corrections, such as tests of normality and adjustment for multiple comparisons
☐	☒	A full description of the statistical parameters including central tendency (e.g. means) or other basic estimates (e.g. regression coefficient) AND variation (e.g. standard deviation) or associated estimates of uncertainty (e.g. confidence intervals)
☐	☒	For null hypothesis testing, the test statistic (e.g. *F*, *t*, *r*) with confidence intervals, effect sizes, degrees of freedom and *P* value noted<br>*Give P values as exact values whenever suitable.*
☒	☐	For Bayesian analysis, information on the choice of priors and Markov chain Monte Carlo settings
☒	☐	For hierarchical and complex designs, identification of the appropriate level for tests and full reporting of outcomes
☒	☐	Estimates of effect sizes (e.g. Cohen's *d*, Pearson's *r*), indicating how they were calculated

Our web collection on statistics for biologists contains articles on many of the points above.

Software and code

Policy information about availability of computer code

Data collection	No software was used
Data analysis	Data were analysed with GraphPad Prism 6.0 Software. The R software was used for calculating linear regressions.

For manuscripts utilizing custom algorithms or software that are central to the research but not yet described in published literature, software must be made available to editors/reviewers. We strongly encourage code deposition in a community repository (e.g. GitHub). See the Nature Research guidelines for submitting code & software for further information.

Data

Policy information about availability of data

All manuscripts must include a data availability statement. This statement should provide the following information, where applicable:

- Accession codes, unique identifiers, or web links for publicly available datasets
- A list of figures that have associated raw data
- A description of any restrictions on data availability

The datasets generated during and/or analysed during the current study are available from the corresponding author on reasonable request.

Field-specific reporting

Please select the one below that is the best fit for your research. If you are not sure, read the appropriate sections before making your selection.

☒ Life sciences ☐ Behavioural & social sciences ☐ Ecological, evolutionary & environmental sciences

For a reference copy of the document with all sections, see nature.com/documents/nr-reporting-summary-flat.pdf

Life sciences study design

All studies must disclose on these points even when the disclosure is negative.

Sample size	For each population doubling, drug treatment, RT-qPCR, ChIP, DNA methylation, and silencing assays three independent experiments were performed, which is the minimum number to estimate the mean and the SD with a good level of confidence to perform statistical tests. We could not afford a larger number of experiments to increase the n value, since the reference population doubling experiments are rather time-consuming (≥ 3 months) and several experiments require large amount of material (i.e. a large number of cells) that is difficult to obtain for cells undergoing senescence. For each test, we indicated all relevant values of the test summary to allow the reader self-evaluating the outcome of the test and our conclusions. For immunofluorescence analysis, which was done assessing the signal in the entire cell volume (3D) (and not just on the cell surface, 2D), a minimum of 30-50 cells and up to 127 cells (e.g. Fig. 4f and Supplementary Fig. 5e,f), from three independent experiments were analysed. For beta-galactosidase cell staining, where only two outputs were considered (positive or negative staining), we analysed up to 4500 cells in sample expected to have low frequency of beta-galactosidase positive staining (e.g. PN16) to estimate this frequency accurately. For other PNs, where the number of beta-galactosidate positive cells was expected to be much higher, assessing 200-400 cells was sufficient to estimate their proportion. We used the SEM to compare the mean of the different groups, and the SD to assess the level of dispersion in the data.
Data exclusions	No data were excluded from analyses.
Replication	Technical replicates have been previously performed for RT-qPCR, WB, immunofluorescence, and culturing experiments to verify that little variation was associated with the technique itself. All experiments were then performed in triplicate, with each unit performed independently. N=3 cell cultures were used for each experiment of population doubling, and each of these three cultures was used for successive assessements (immunoflorescence, WB, RT-qPCR, ChIP, DNA methylation assys). Silencing experiments were also done in three independent experiments, using for each set of experiment two independently silenced clones (1 and 2, as reported). Each set of experiments (n=3) involving population doubling was assessed independently. Indeed, comparison among passage numbers (PN) from different sets of experiments is not recommended (for instance cells frozen at a given passage and then cultured after thawing result in early appearance of senescence markers compared to cells with the same nominal PN but continuously cultured (no freezing)). For this reason, independent assessment was also done for RT-qPCR, WB, and other experiments with material derived from each set of population doubling experiments. Extra cell pellets have been frozen and extra slides with fixed cells for immunofluorescence studies have been kept from each experiment to perform further tests required for the revision of the manuscript.
Randomization	Randomization was not relevant to our study since we used a single cell type, in the presence and in the absence of a treatment (drug, silencing), or in longitudinal conditions (successive culturing).
Blinding	Blinding was not applied to these experiments since the same experimenter was in charge of preparing and analysing the samples. All steps leading to results were documented and raw data were available at any time to the other co-authors

Reporting for specific materials, systems and methods

We require information from authors about some types of materials, experimental systems and methods used in many studies. Here, indicate whether each material, system or method listed is relevant to your study. If you are not sure if a list item applies to your research, read the appropriate section before selecting a response.

Materials & experimental systems

n/a	Involved in the study	
☐	☒	Antibodies
☐	☒	Eukaryotic cell lines
☒	☐	Palaeontology
☒	☐	Animals and other organisms
☒	☐	Human research participants
☒	☐	Clinical data

Methods

n/a	Involved in the study	
☒	☐	ChIP-seq
☒	☐	Flow cytometry
☒	☐	MRI-based neuroimaging

Antibodies

Antibodies used

Alexa Fluor 488 α-mouse IgG; Thermo Fisher Scientific; Cat#A-11029, RRID: AB_2534088
Alexa Fluor 488 α-rabbit IgG; Thermo Fisher Scientific; Cat#A-11034, RRID: AB_2576217
Alexa Fluor 555 α-mouse IgG; Thermo Fisher Scientific; Cat#A-21425, RRID: AB_2535846
Alexa Fluor 555 α-rabbit IgG; Thermo Fisher Scientific; Cat#A-21430, RRID: AB_2535851
CF770 Goat α-Mouse IgG; Biotium; Cat#20077, RRID:AB_10559194
CF770 Goat α-Rabbit IgG; Biotium; Cat#20078, RRID:AB_10563034
Chicken Polyclonal α-GFP; Abcam; Cat#ab13970, RRID: AB_300798
hFAB Rhodamine α-GAPDH ; Bio-Rad; Cat#12004168
hFAB Rhodamine α-β-Tubulin; Bio-Rad; Cat#12004166
HRP Goat α-Mouse IgG; Thermo Fisher Scientific; Cat# 31430, RRID:AB_228307
HRP Goat α-Rabbit IgG; Thermo Fisher Scientific; Cat# 31460, RRID:AB_228341
Mouse Monoclonal α-5-methlycytosine (clone 33D3); Merck; Cat#MABE146, RRID:AB_10863148

	Mouse Monoclonal α-ATP Synthase beta (clone 3D5AB1); Thermo Fisher Scientific; Cat#A-21351, RRID:AB_221512<br>Mouse Monoclonal α-CDKN2A/p16INK4a (clone2D9A12); Abcam; Cat#ab54210, RRID:AB_881819<br>Mouse Monoclonal α-CSB (clone 1CSB-3H8); Euromedex; Cat#CSB-3H8<br>Mouse Monoclonal α-Histone H2A.XpSer139; Millipore; Cat#05-636, RRID:AB_309864<br>Mouse Monoclonal α-p21; BD Biosciences; Cat#556430, RRID:AB_396414<br>Mouse Monoclonal α-p53 (clone DO-1); Santa Cruz Biotechnology; Cat#sc-126, RRID:AB_628082<br>Mouse Monoclonal α-PCNA (clone PC10); Santa Cruz Biotechnology; Cat#sc-56, RRID:AB_628110<br>Mouse Monoclonal α-phospho-ATM (pS1981); Rockland Immunochemicals; Cat#200-301-400, RRID:AB_217868<br>Rabbit Monoclonal α-CSA; Abcam; Cat#ab137033, RRID:AB_2783825<br>Rabbit Monoclonal α-Cyclin A2; Abcam; Cat#ab181591<br>Rabbit Polyclonal α-53BP1; Novus ; Cat#NB100-304, RRID:AB_10003037<br>Rabbit Polyclonal α-CSB; Bethyl; Cat#A301-345A, RRID:AB_937849<br>Rabbit Polyclonal α-GAPDH (clone FL-335); Santa Cruz Biotechnology; Cat#sc-25778, RRID:AB_10167668<br>Rabbit Polyclonal α-GFP; Rockland Immunochemicals; Cat#600-401-215, RRID:AB_828167<br>Rabbit Polyclonal α-Histone H3; Abcam; Cat#ab1791, RRID:AB_302613<br>Rabbit Polyclonal α-Histone H3ac; Millipore; Cat#06-599, RRID:AB_2115283<br>Rabbit Polyclonal α-HTRA2 ; Sigma-Aldrich; Cat#HPA027366, RRID:AB_1851301<br>Rabbit Polyclonal α-HTRA3 ; Sigma-Aldrich; Cat#HPA021187, RRID:AB_1851311<br>Rabbit Polyclonal α-POLG1 (clone H-215) ; Santa Cruz Biotechnology; Cat#sc-48815, RRID:AB_2166864<br>Rabbit Polyclonal α-TOMM22; Sigma-Aldrich; Cat#HPA003037, RRID:AB_1080329<br>StarBright Blue 700 Goat α-Mouse IgG; Bio-Rad; Cat#12004159<br>StarBright Blue 700 Goat α-Rabbit IgG; Bio-Rad; Cat#12004161, RRID:AB_2721073<br>Total OXPHOS Human WB Antibody Cocktail; Abcam; Cat#ab110411, RRID:AB_2756818<br>α-Normal Rabbit IgG; Santa Cruz Biotechnology; Cat#sc-2027, RRID:AB_737197
Validation	All antibodies above were validated by the producer (relevant publications were cited in the individual technical sheet) for the species used here (human; with the exception of α-GFP (Abcam, ab13970) that is ectopically expressed), as well as the specific application for which they have been used in our study (IF, WB, and ChIP).

Eukaryotic cell lines

Policy information about cell lines

Cell line source(s)	IMR-90 primary lung fibroblasts were purchased from ATCC (ATCC®-CCL186TM).
Authentication	No further autentication was done; cells were thawed from the original commercial vial.
Mycoplasma contamination	IMR-90 cells were puchased from ATCC, aliquoted and kept in liquid nitrogen before use. We tested for the presence of mycoplasma cells after the first aliquoting and cells at PN18, which includes several consecutive passage numbers in the present expeirments. At PN18 we tested both the culture medium of confluent cells kept in culture for 48h (to maximise the presence of mycoplams, if any), and also assessed the cell pellet (2 million of cells), with PCR amplification of mycoplasma-specific sequences (primers: MGSO: TGCACCATCTGTCACTCTGTTAACCTC and GPO1: ACTCCTACGGGAGGCAGCAGTA, as described in Pruckel et al, 1995, Detection by polymerase chain reaction of all common Mycoplasma in a cell culture facility. Pathobiology 63:9-11;, PMID 7546276). As positive control we used a stock of validated mycoplasma containing supernatant that results in huge PCR amplification under our experimental conditions. The result was negative for all tested material.
Commonly misidentified lines<br>(See ICLAC register)	N/A
